# Evaluation of a Polyherbal Formulation on the Management of Migraine Headaches due to Functional Dyspepsia: A Double-Blind, Randomized, Placebo-Controlled Clinical Trial

**DOI:** 10.1155/2022/9872933

**Published:** 2022-12-03

**Authors:** Majid Anushiravani, Seyed Mousal-Reza Hosseini, Karim Nikkhah, Shabnam Niroumand, Ali Reza Derakhshan, Roshanak Salari, Ali Gholampour, Vahid Reza Askari

**Affiliations:** ^1^Department of Persian Medicine, School of Persian and Complementary Medicine, Mashhad University of Medical Sciences, Mashhad, Iran; ^2^Department of Gastroenterology, School of Medicine, Mashhad University of Medical Sciences, Mashhad, Iran; ^3^Department of Neurology, Ghaem Medical Centre, Mashhad University of Medical Sciences, Mashhad, Iran; ^4^Department of Community Medicine, Faculty of Medicine, Mashhad University of Medical Sciences, Mashhad, Iran; ^5^Department of Pharmaceutical Sciences in Persian Medicine, School of Persian and Complementary Medicine, Mashhad University of Medical Sciences, Mashhad, Iran; ^6^International UNESCO Center for Health-Related Basic Sciences and Human Nutrition, Mashhad University of Medical Sciences, Mashhad, Iran; ^7^Iran Applied Biomedical Research Center, Mashhad University of Medical Sciences, Mashhad, Iran

## Abstract

A holistic concept based on traditional Persian medicine (TPM) describes a headache with a gastrointestinal (GI) origin (gastric-headache). Although the neurological manifestations of this headache are similar to those of other headaches, its etiology is different. Considering its simultaneous effects on the brain and GI system, a formulation was designed based on this concept. This study aimed to determine the safety and efficacy of the designed formulation on migraine headache (MH) associated with functional dyspepsia (FD). A total of 75 diagnosed cases of MH patients with concurrent FD were randomly divided equally into 3 groups: (i) the polyherbal formulation, sodium valproate (VPA), and amitriptyline group, (ii) VPA, amitriptyline, and polyherbal formulation placebo group, and (iii) the polyherbal formulation and VPA placebo group. The primary outcomes, including frequency, duration, and severity of MH attacks, were measured at baseline and weeks 4, 8, and 12. However, secondary outcomes, including the Headache Impact Test 6 (HIT-6) Questionnaire and Parkman's score, were evaluated at baseline and end of treatment. The frequency, duration, and severity of migraine (*P* < 0.001 for all cases), HIT-6 (*P* < 0.001 for all cases), and FD (*P* < 0.001 for all cases) scores at the end of treatment showed a significant decrease in the 3 groups compared to the baseline. However, the differences in those variables between the 3 groups were not significant at the end of the study. The polyherbal formulation alone may improve the symptoms of migraine patients and other groups. This effect could be due to improving digestion and FD in migraine patients.

## 1. Introduction

The International Headache Society (IHS) has classified headaches into primary and secondary types. Primary headaches include migraine, tension-type, and cluster headaches [1]. Migraine headache (MH) is described as a chronic neurovascular disorder in the brain, characterized by unilateral throbbing pain [2, 3]. It is recognized as one of the top 10 debilitating diseases worldwide. According to the burden of disease study in 2013, this disorder was ranked the sixth cause of disability, negatively impacting the individual's quality of life, occupation, social activities, and education [3, 4].

On the other hand, functional dyspepsia (FD) is a relapsing and remitting disorder characterized by upper abdominal discomfort, early satiety, postprandial abdominal distension, nausea, vomiting, and anorexia, according to ROME III and Parkman's criteria [5, 6]. The prevalence of FD ranges between 5% and 11% worldwide [7] and is estimated at 32.1% in Iran [8]. Also, the prevalence of MH is 12% in the general population, and women are 3 times more susceptible to this disease than men [9].

Interestingly, the prevalence of dyspepsia in Lankarani et al. study's was about 32.1% [8]. Moreover, Iranian studies have indicated that dyspepsia's prevalence ranges from 2.2% to 29.9% [10, 11]. Perhaps, the explanations for these vast differences may be due to the fact that some of these studies did not use ROME Diagnostic Criteria for Functional Gastrointestinal Disorders (FGIDs). Furthermore, Lankarani et al. also reported that 33.3% of the irritable bowel syndrome (IBS) group had MHs [8]. This association could be explained by a higher prevalence of anxiety/depression in both groups and may result from visceral hyperalgesia [12, 13]. Indeed, it could be postulated that migraine patients with FD may experience higher hypersensitivity to gastric distention and lower tolerance to food than healthy people [14]. Further studies also revealed that comorbid reflux in migraine patients ranged from 22% to 42.6% [15, 16]. It has also been reported the correlation between gastric reflux and migraine attack [14]. Additionally, autonomic nervous system (ANS) dysfunction can play a crucial role in the etiology of both gastric reflux and migraine [17, 18]. Noteworthy, abnormal visceral hypersensitivity may have an essential role in dyspeptic and migraine patients [19, 20]. Furthermore, several neuropeptides are involved in dyspeptic and migraine patients, such as the neuropeptide calcitonin gene-related peptide (CGRP), which is increased during migraine attacks and can also cause dyspepsia [21].

Genetic, hormonal, immune, neuronal, and environmental factors are implicated in susceptibility to MH [22, 23]. In addition, the disturbance in the gut-brain axis (GBA) and other disorders, such as menstrual cycle disorders, infections, homeostasis disorders, and gut dysbiosis, has been reported, playing vital roles in the physiopathology of MH [3, 12, 24, 25]. Furthermore, major FGIDs associated with MH include gastroesophageal reflux (GERD), inflammatory bowel disease (IBD), FD, IBS, gastroparesis, celiac disease, and *Helicobacter pylori* infection [8, 12, 26–28]. However, the physiopathology of MH is complex and not well-defined [29].

Based on previous studies, the central nervous system (CNS) is linked to the gastrointestinal (GI) system in various ways. The CNS interacts with GI and the enteric nervous system (ENS) [30]. This bidirectional communication is referred to as GBA [15, 31, 32]. It involves interactions with signals from the gut to the brain and from the brain to the gut by neural, endocrine, and immune pathways. The GBA interactions' mechanisms involve neuro-immuno-endocrine mediators [30]. Recent research has described the importance of gut microbiota in these interactions [31]. Therefore, a better understanding of these interactions may offer new targeted therapies.

Several preventive strategies are used for MH, including antiepileptic drugs, such as sodium valproate (VPA), tricyclic antidepressants, anti-inflammatory drugs, and beta-blockers [3, 33]. However, although current treatments can control the symptoms in some patients, there are nonresponsive multiple cases to treatment. Also, complications such as somnolence, hypotension, tremor, weight gain, constipation, and polycystic ovarian disease [34] prompt researchers to explore more effective and less complicated medications. Therefore, complementary and alternative medicines (CAMs), especially traditional medicine approaches and medicinal plants, have gained popularity among patients [35].

Several studies have evaluated CAM use among patients with specific chronic diseases, including diabetes, cancer, IBS, gingivitis, and cardiovascular diseases, with evidence suggesting high CAM use among patients with chronic illnesses [36–38]. A holistic concept based on traditional Persian medicine (TPM) describes a type of headache with a GI origin (gastric-headache) [28, 39–42]. In fact, the origin of this type of headache lies in the GI tract. Thus, according to this concept and the current medical literature, treatment plans for patients with headaches due to FGID should be focused on the GI system [43]. A formulation was designed based on this concept considering its simultaneous effects on the brain and GI. Thus, in the current study, we aimed to evaluate the effectiveness and safety of a polyherbal formulation in MH management associated with FD and compare the results with standard medication.

## 2. Materials and Methods

### 2.1. Preparation and Standardization of Polyherbal Formulation and Placebo

The constituents of the polyherbal formulation consist of *Phyllanthus emblica L*. (Amla), *Rosa damascena* Mill *L*. (Damask rose), *Coriandrum sativum* (coriander), and *Zingiber officinalis* (ginger). Next, the formulations were powdered using a grinder and filtered through a 100-mesh stainless steel screen to obtain isodiametric particles. The final powder was used to prepare the capsules. The capsules were prepared by the School of Pharmacy, Mashhad University of Medical Sciences, Mashhad, Iran, containing 750 mg of the polyherbal formulation, including 150 mg of Damask rose petal and 150 mg of coriander seeds, 250 mg of ginger rhizomes, and 200 mg of dried amla fruit. The capsules were packaged in plastic boxes containing 90 capsules. The standardization of the polyherbal formulation was based on total phenol. Gallic acid in the hydroalcoholic extract constituted 229 mg/g of the crude extract. Also, we used 200-mg VPA tablets (Depakine®, Sanofi Co., France).

In the current study, we considered 2 types of placebo prepared by the School of Pharmacy, Mashhad University of Medical Sciences, Mashhad, Iran. The placebo tablets contained 60% microcrystalline cellulose, 20% lactose, 19.5% starch, and 0.5% magnesium stearate [44], similar in size and shape to Depakine®. In addition, the placebo capsules also contained microcrystalline cellulose, identical to the polyherbal formulation capsules [44].

### 2.2. Study Design and Case Selection

This study was conducted in 2 educational hospitals (Qaem and Imam Reza hospitals) affiliated with Mashhad University of Medical Sciences, Mashhad, Iran, from May 2017 to August 2018. After screening and selecting eligible subjects, the baseline information, demographic characteristics (age, sex, occupation, educational level, marital status, and body mass index (BMI)), type of migraine (with/without aura), and family and personal history of migraine were documented. Three main aspects of MH (frequency, duration, and severity) were recorded to assess the attacks.

This study was approved by the Ethics Committee of Mashhad University of Medical Sciences (IR.MUMS.AC.REC.1350.58). All patients were informed about the study protocol, and signed informed consent was obtained before entering the study. This randomized clinical trial was registered on the Iranian registry of clinical trials website (IRCT20170317033107N3).

This randomized, double-blind clinical trial was performed on patients with MH associated with FD. The selected patients met the diagnostic criteria for MH according to the International Classification of Headache Disorders-III*β* (ICHD-III *β*) [1] and ROME III and Parkman's criteria for FD in the past 6 months [45]. A 12-item checklist was completed for the patients to evaluate the comorbidity of MH with FD.

Furthermore, the Headache Impact Test 6 (HIT-6) was also performed for the patients at the beginning and end of the study. This questionnaire contains 6 questions, with a minimum score of 36 and a maximum score of 72. Higher scores indicate the more significant impact of headache on disabling pain experience, daily function, social function, vitality, cognitive function, and psychological distress [46].

Noteworthy, all diagnoses are based on specific criteria by a neurologist and gastroenterologist. Additionally, to determine the effects of the interventions in this study, patients were advised not to change their medications (both for migraine and dyspepsia).

To determine possible side effects, routine laboratory tests, including complete blood cell count (CBC), fasting blood sugar (FBS), triglyceride (TG), cholesterol, aspartate aminotransferase (AST), alanine aminotransferase (ALT), and total and direct bilirubin, were carried out. Since all tests were normal both before and after the interventions, the data are not shown.

### 2.3. Inclusion and Exclusion Criteria

All Iranians of Persian ethnicity aged 18–55 years diagnosed with MH (based on the ICHD-III *β* criteria) [47] and concurrent FD (based on ROME III and Parkman's criteria) by a neurologist and gastroenterologist were included in this study. Moreover, the patients should have 18 < BMI < 25 kg/m^2^ without the following criteria during the study: (1) bile reflux, cholelithiasis, or steatohepatitis, (2) uncontrolled hypertension, diabetes, GI cancer, or peptic ulcer, (3) pregnancy or lactation, (4) occurrence or diagnosis of any hepatic, renal, cardiac, pulmonary, systemic, or psychological diseases, (5) neurological diseases other than MH during the study, and (6) history of using nitrate-containing medicines. Based on the exclusion criteria, we aligned the study's patients using new migraine cases with FD. Therefore, the medications were considered the same for both groups.

### 2.4. Intervention Groups

There were 3 intervention groups in this study that received treatment as follows: d how to use.

**Group 1:** The polyherbal formulation capsule, 1,500 mg (2 capsules of 750 mg) 3 times a day + Depakine® tablet, 200 mg, twice a day + Amitriptyline tablet, 10 mg, once a day.

**Group 2:** Depakine® tablet, 200 mg, twice a day + Amitriptyline, 10 mg, once a day + the polyherbal formulation placebo capsule 3 times a day.

**Group 3:** The polyherbal formulation capsule, 1,500 mg (2 capsules of 750 mg) 3 times a day + Depakine® placebo tablet twice a day.

The duration of the intervention was 12 weeks in this study. During this period, the patients were followed up to determine the frequency, duration, and severity of MH attacks in weeks 4, 8, and 12. HIT-6 was completed, and ROME III and Parkman's criteria were evaluated both at baseline (week 0) and end of week 12. The possible adverse effects of medications were also monitored during the intervention using laboratory tests. At the end of the clinical trial and 6 weeks after the intervention termination (week 18), the patient's satisfaction with the treatment, symptom relief, and relapse was surveyed using a Likert scale. A specialist collected all data using a handmade preformed checklist of criteria. Based on their medication compliance, all medications were delivered to patients every 15 to 30 days.

The patients also received a dietary guide pamphlet on abstinence orders and behavioral advice, which are described below:

*Abstinence orders*: Restrictions on some types of foods, including mucus-producing (e.g., soft-wheat bread, cereal, and bananas), flatulent, hard-to-digest, and heavy meals (e.g., spaghetti); some dairy products according to TPM and current nutrition guidelines; and especially some high-FODMAP (fermentable oligo-, di-, monosaccharides, and polyols) foods (e.g., broccoli, garlic, onion, pasta, watermelon, yogurt, fresh cheese, and mushrooms) [48].

*Behavioral advice*: Avoid drinking cold water with an empty stomach or after intensive physical activity and exercise [49, 50], avoid overeating, and keep a regular meal [51].

The patients were required to complete a self-report checklist regarding their adherence to the guidelines, their abstinence, and the medicine used during the course of treatment. There are no definitive guidelines for establishing consensus regarding the current study and the cutoff. While some researchers have suggested that 51% agreement among respondents can indicate consensus regarding the cutoff, others have adopted more stringent levels. We defined consensus as 75% as the cutoff or more responses falling within a 2-point bracket on a response scale, according to Clyne et al. study's [52].

### 2.5. Sample Size Estimation

Based on a pilot study on 15 patients with migraine-associated FD (indicating a 65% improvement in migraine attacks), as well as the sample size of similar studies (including studies by Fazljou et al. and Ghorbanifar et al.) [53], we recruited 75 patients and randomly divided them into 3 groups.

### 2.6. Randomization and Blinding

A total of 75 participants were randomly assigned a number using SPSS software from 1 to 75 and divided into 3 columns (A, B, and C). The subjects were placed in one of the groups according to their order of entering the study. Noteworthy, this study was a double-blind clinical trial, and patients and researchers were unaware of the treatment groups.

### 2.7. Statistical Analysis

The final clinical and paraclinical data were analyzed using SPSS version 16. The characteristics of each group were described by descriptive methods, including central tendency and dispersion, according to the study's objectives and studied variables. The nonparametric Friedman's test was used to compare quantitative variables (i.e., frequency, duration, and severity of MH attacks) in the 3 groups from week 0 to week 12. The nonparametric Kruskal–Wallis's test was also used to compare mean differences between the groups at the beginning and end of the study. Moreover, Spearman's nonparametric test was performed to investigate the correlations of frequency, duration, and severity of MH attacks with severe FD. In all calculations, *P* values less than 0.05 were considered statistically significant.

## 3. Results

### 3.1. Baseline Characteristics

**Figure 1:**
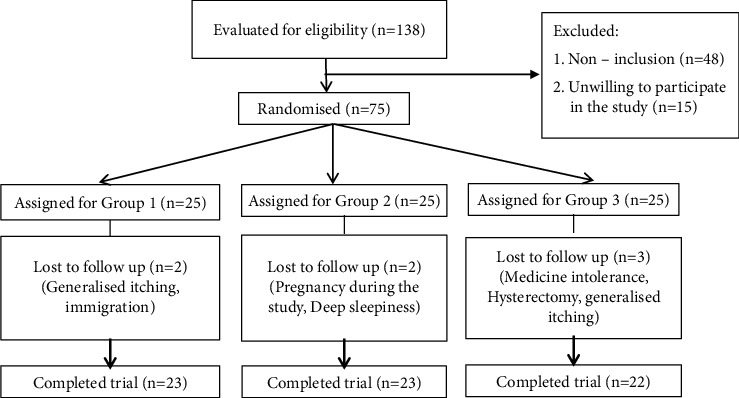
Flowchart of the study. **Group 1:** the polyherbal formulation capsule^*∗*^ 1500 mg (two capsules of 750 mg) three times a day, + Depakine® tablet 200 mg twice a day + Amitriptyline tablet 10 mg once a day; **Group 2:** Depakine® tablet 200 Mg twice a day + Amitriptyline 10 mg once a day + the polyherbal formulation placebo capsule three times a day; **Group 3:** the polyherbal formulation capsule 1500 mg (two capsules of 750 mg) three times a day + Depakine® placebo tablet twice a day.

A total of 75 MH patients with concurrent FD participated in this study and were randomly divided into 3 intervention groups.  Figure 1 presents the CONSORT (Consolidated Standards of Reporting Trials) flow diagram of the study. The baseline characteristics are summarized in Table 1. There was no significant difference in terms of age, sex, marital status, and educational level between the 3 groups.

### 3.2. The Effects of the Interventions on the Frequency, Duration, and Severity of MH Attacks

**Figure 2:**
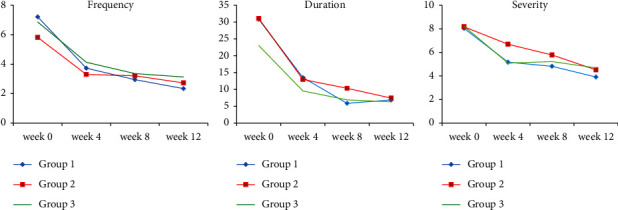
Frequency, duration and severity of MH attacks (per month) in three groups throughout the study period from week 0 to week 12. **Group 1:** the polyherbal formulation capsule^*∗*^ 1500 mg (two capsules of 750 mg) three times a day, + Depakine® tablet 200 mg twice a day + Amitriptyline tablet 10 mg once a day; **Group 2:** Depakine® tablet 200 Mg twice a day + Amitriptyline 10 mg once a day + the polyherbal formulation placebo capsule^*∗*^ three times a day; **Group 3:** the polyherbal formulation capsule 1500 mg (two capsules of 750 mg) three times a day + Depakine® placebo tablet twice a day.

As mentioned in Table 2, the reduction of frequency, duration, and severity of MH attacks were significant in the 3 groups compared to the beginning of the study (*P* < 0.001 for all cases). However, the intergroup analysis showed no significant difference between the groups regarding the frequency, duration, and severity of MH attacks (*P*=0.61, *P*=0.25, and *P*=0.30, respectively; Table 2).  Figure 2 indicates the mean frequency (a), duration (b), and severity (c) of MH attacks at weeks 0, 4, 8, and 12 after the intervention. It was found that the slope decreased for the variables, especially during the first 4 weeks of treatment.

### 3.3. The Effects of the Interventions on HIT-6 Scores

Table 3 represents the subjects' HIT-6 scores at weeks 0 and 12. At the end of the study, the results indicated that the intervention was effective in improving the quality of life, occupational status, and educational level in the 3 groups, and the difference was statistically significant compared to the beginning of the study (*P* < 0.001 for all cases; Table 3). However, there was no significant difference between the groups at the beginning and end of the study (*P*=0.88 and *P*=0.87, respectively; Table 3). Also, the intervention could improve the HIT-6 scores from severe to substantial (impact the grades of HIT-6: little/none, moderate, substantial, and severe).

Moreover, the results indicated that the intervention could significantly reduce the score of FD in all 3 groups compared to the beginning of the study (*P* < 0.001 for all cases; Table 3). Although Kruskal–Wallis's test showed no statistically significant differences between the 3 groups regarding the FD score at the end of the study (*P*=0.06; Table 3), the reduction of the FD score from week 0 to week 12 was significantly higher in group 1 vs. group 2 (*P*=0.002; Table 3) and group 3 vs. group 2 (*P*=0.01; Table 3).

### 3.4. Correlation of Duration, Severity, Frequency of MH Attacks, and HIT-6

According to Spearman's Rho test, Table 4 indicates the correlation coefficients at the end of the intervention (week 12). The results showed that the duration of MH in group 3 (*ρ*  = 0.55, *P*=0.007; Table 4), the severity of MH in groups 2 and 3 (*ρ*  = 0.45, *P*=0.03 and *ρ*  = 0.57, *P*=0.005, respectively; Table 4), and HIT-6 score in groups 2 and 3 (*ρ*  = 0.47, *P*=0.02 and *ρ*  = 0.66, *P*=0.001, respectively; Table 4) had significant positive correlations with the score of FD. In other words, a decrease in the score of FD indicated a substantial reduction in the mentioned variables (Table 4).

### 3.5. The Possible Clinical Adverse Effects of the Polyherbal Formulation

Moreover, the patient's satisfaction with treatment, symptom relief, and relapse was surveyed based on a Likert scale. After 6 weeks of the intervention, 67% of the subjects agreed or strongly agreed with the treatment process; however, 23% were neutral, and 10% reported relapse. Also, 4 patients developed drug-related complications, including 2 patients with generalized itching, 1 with excessive sleepiness, and 1 with polyherbal formulation intolerance.

## 4. Discussion

This study was designed based on a TPM concept. According to this concept, a disease of a specific organ may originate from another organ. For instance, the stomach has a compound tissue composed of interwoven nerves interconnected with the brain, which can substantially impact the brain. From the TPM perspective, the body is an integrated system where different organs are interconnected [28, 39]. TPM manuscripts describe a form of headache that has a GI origin. This type of headache does not primarily occur due to cerebral disorders but arises from the stomach's structural and functional disorder [43]. Based on the teachings of Avicenna (981–1037 AC), some theories can explain this disorder, including the theory of constitutional continuity [54], stating that some organs are connected through histologic similarities.

Recent anatomic-physiological studies have shown that the vagus nerve and ENS can also play essential roles in the interconnection of organs [55]. On the other hand, brain disorders affect stomach activity and impair digestion. Supporting evidence for the gastrocerebral pathogenesis of migraine is cyclic vomiting syndrome, which is highly common in migraine patients, especially in children [1, 56]. In addition, many migraine patients experience nausea and vomiting during a headache episode, whereas emptying the stomach significantly reduces headaches [57].

A growing body of evidence strongly suggests the gut-brain interaction. It has been shown that the bidirectional GBA axis sends signals from the ENS to the brain through nerves, the endocrine system, and the immune system. Previous research suggests that the gut microbiota is connected to GBA and modulates this axis [30]. Also, dysbiosis affects CNS, ENS, immune system, and epithelial barrier permeability and may lead to malabsorption, poor digestion, and, finally, headache [58, 59].

According to several studies, clinical features such as loss of appetite, poor digestion, bloating, belching, rumbling, upset stomach, and nausea before migraine attacks can raise clinical suspicion for gut-induced MH [49]. However, more than 60% of the participants reported that their headaches had deteriorated by hunger, GERD, and eating flatulent food in our study. Therefore, our central hypothesis was based on the notion that migraine patients with FD could be treated by improving dyspepsia.

The polyherbal formulation used in the present study consisted of 4 compounds with beneficial effects on 4 aspects of gastric function, leading to a reduction of gas production and accumulation in the stomach, an increase of the lower esophageal sphincter tonicity, improvement of the antral motility and gastric emptying, and improvement of FD symptoms [42, 60–65]. Additionally, these changes result in decreased gastric heaviness, belching, and abdominal distention and prevent GERD. Therefore, using these compounds can play a prophylactic role in the occurrence of MH attacks, reduce the frequency and duration of episodes, and alleviate the severity of attacks.

As reported in several previous studies, the polyherbal formulation components used in this study seem to have therapeutic effects on headaches (Table 5). For instance, *C. sativum* positively affected MH by reducing the frequency, duration, and severity of attacks compared to the control group [53]. Also, a traditional herbal formulation containing *P. emblica* had similar protective effects on MH during a 3-month intervention [66]. Also, a clinical trial showed that a 3-month use of the *Z. officinalis* extract significantly decreased the frequency of migraine attacks [42, 67]. However, the effect of ginger on headache severity was not significantly different from that of placebo or sumatriptan [68]. In a before-after study, a mixture of *R. damascena* and honey effectively reduced the frequency of migraine attacks [69]. Nevertheless, it was ineffective in decreasing the duration or severity of the episodes [69].

In the current study, we considered Depakine® as a routine medication for MH. Moreover, Depakine® effectively decreased the duration of MH, according to previous studies [70]. Our results showed that the combination of Depakine® with the polyherbal formulation had more significant effects on reducing the duration of MH compared to Depakine® alone. The highest reduction occurred in the first 4 weeks of the intervention. Although the positive impact of some of our drug components, such as Damask rose and ginger, in reducing headache severity has not been studied previously, our results showed that the polyherbal formulation components could significantly reduce the headache severity at the end of treatment in the 3 groups.

Numerous studies have shown the association of GIDs (e.g., IBD, gastroparesis, hepatobiliary disease, celiac disease, and dysbiosis) with the occurrence of migraine. The possible mechanisms include response to chronic inflammation and the presence of vasoactive peptide mediators (e.g., calcitonin gene-related peptide [71], substance P, and neurokinin released from trigeminal fibers), leading to the activation of peripheral nerve sensory terminals in the meningeal brain structure; other possible mechanisms include gut microbiota disorders, which play an important role in modulating the immune system, and finally ANS disorders. In addition, recent physiopathological studies have reported the risk of microbiome dysfunction, duodenal inflammation, increased permeability, and dysbiosis in patients with FD [8, 12, 25, 55, 72–74]. Thus, besides the effects of the polyherbal formulation components on the brain, they also have shown some GI activities, which may be responsible for their impact on MH (Table 5).

Based on the evidence discussed in TPM and current phytopharmacological studies, the possible mechanisms contributing to the efficacy of the poly-herbal formulation in migraine treatment may be the increased tonicity of the gastroesophageal junction, improvement of the gastric emptying rate, reduction of gas production and decomposition in the gut, and prevention of gas effects on the brain. Overall, a decrease in FD symptoms can decrease the rate of MH attacks.

As a limitation of the current study, the study was scientifically and ethically approved in 2017, the final version of the ICHD was ICHD-3*β* [47], and we designated the survey according to the parameters. One of the changes introduced from ICHD-3*β* to ICHD-3 was a change to the diagnostic criteria of migraine with aura and migraine with typical aura, with the aim of better differentiating migraines from transient ischemic attacks [75]. However, as we followed the patients, none had experienced transient ischemic attacks or worsened ones. It is very difficult to manage patients in clinical trial studies; as a result, some patients need more control and supervision to receive and consume drugs. Therefore, we had to deliver the medicines between 15 and 30 days by case to overcome the issue. However, another limitation of this study method is that it is not routine and may provide bias in the blinding. Furthermore, the patient's characteristics were measured during the first 4 weeks (basic evaluation). Eventually, we tried to overcome the issue using more rigorous statistical tests.

## 5. Conclusion

**Figure 3:**
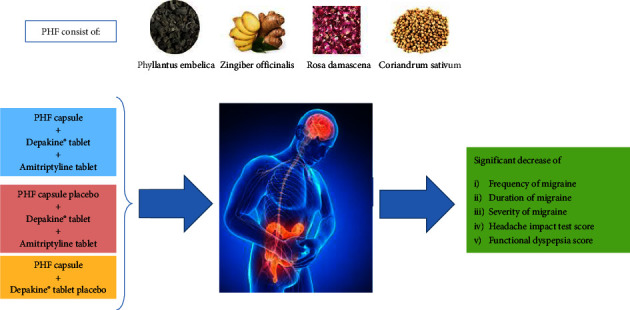
A schematic overview of the current study. ^*∗*^PHF is an abbreviation for polyherbal formulation.

Based on the results, we can conclude that using the polyherbal formulation and dietary abstinence may positively affect the frequency, duration, and severity of MH. Also, the polyherbal formulation can improve FD in combination with dietary abstinence and VPA. Therefore, the polyherbal formulation has remarkable effects on FD and can be used as an adjuvant therapy with VPA for MH (Figure 3). However, further studies with a larger sample size are required to confirm the efficacy of the poly-herbal formulation.

## Figures and Tables

**Table 1:** Demographic characteristics of the participants in the present study in an overview.

Variables		Group 1	Group 2	Group 3	Total
*Gender; N (%)*	Female	18 (78.2%)	19 (82.6%)	15 (68.1%)	52 (76.4%)
	Male	5 (21.8%)	4 (17.4%)	7 (31.9%)	16 (23.6%)
*Marital status; N (%)*	Single	6 (26.1%)	7 (30.4%)	3 (13.6%)	16 (23.5%)
	Married	17 (73.9%)	16 (69.6%)	19 (86.4%)	52 (72.5%)
*Type of migraine; N (%)*	Without Aura	18 (78.2%)	17 (73.9%)	18 (78.2%)	53 (77.9%)
	With Aura	6 (21.8%)	5 (26.1%)	4 (21.8%)	15 (12.1%)
*Age (year); (Mean ± SD)*	Male/Female	35 ± 10.6	37 ± 7.7	43 ± 13.3	38.3 ± 10.32
*Weight (Kg); (Mean ± SD)*	Male/Female	70 ± 18.8	69 ± 18.9	74 ± 17	71 ± 18.23
*Education; N (%)*	Under Diploma	11 (47.8%)	11 (47.8%)	8 (36.4%)	30 (44.2%)
	University/College	12 (52.2%)	12 (52.2%)	14 (63.6%)	38 (55.8%)
*Occupation; N (%)*	Housewife	5 (21.7%)	7 (30.4%)	5 (22.7%)	17 (25%)
	Employee	17 (73.9%)	16 (69.5%)	16 (72.7%)	49 (72%)
	Unemployed	1 (4.3%)	0 (0%)	1 (4.5%)	2 (2.9%)
*Family history of migraine*	Male/Female	15 (65.2)	20 (87%)	17 (77.3%)	52 (76.4%)

**Group 1:** the polyherbal formulation capsule^*∗*^ 1500 mg (two capsules of 750 mg) three times a day,  + Depakine® tablet 200 mg twice a day + Amitriptyline tablet 10 mg once a day; **Group 2:** Depakine® tablet 200 Mg twice a day + Amitriptyline 10 mg once a day + the polyherbal formulation placebo capsule^*∗*^ three times a day; **Group 3:** the polyherbal formulation capsule 1500 mg (two capsules of 750 mg) three times a day + Depakine® placebo tablet twice a day.

**Table 2:** The effects of different interventions on the levels of frequency, duration and severity of the disease.

Group No	Variable	Week 0	Week 4	Week 8	Week 12	*P*-value^*∗*^	Difference in week 0 and 12
**1**	*Frequency*	7.21 ± 4.65	3.73 ± 2.47	2.95 ± 1.77	2.34 ± 1.11	*P* < 0.001	4.86 ± 4.37
**2**		5.82 ± 2.60	3.30 ± 1.76	3.21 ± 2.15	2.73 ± 1.68	*P* < 0.001	3.08 ± 2.44
**3**		6.86 ± 3.80	4.13 ± 2.47	3.36 ± 1.52	3.13 ± 1.48	*P* < 0.001	3.72 ± 3.39
							*P* ^*∗∗*^=0.61

**1**	*Duration*	30.86 ± 16.27	13.52 ± 12.84	5.86 ± 3.75	6.86 ± 5.48	*P* < 0.001	24 ± 15.81
**2**		31.08 ± 20.96	12.95 ± 8.33	10.34 ± 6.59	7.43 ± 5.40	*P* < 0.001	23.65 ± 18.73
**3**		23.04 ± 15.42	9.54 ± 6.92	6.90 ± 5.19	6.31 ± 5.29	*P* < 0.001	16.72 ± 13.58
							*P* ^*∗∗*^=0.25

**1**	*Severity*	8.04 ± 1.71	5.71 ± 2.05	4.48 ± 2.56	3.91 ± 1.31	*P* < 0.001	4.13 ± 2.13
**2**		8.17 ± 0.98	6.69 ± 3.99	5.78 ± 4.16	4.52 ± 2.23	*P* < 0.001	3.65 ± 2.49
**3**		8.22 ± 1.30	5.09 ± 2.02	5.22 ± 2.59	4.68 ± 2.58	*P* < 0.001	3.54 ± 2.73
							*P* ^*∗∗*^=0.30

Data were presented as mean and standard deviation (SD). ^*∗*^*P* value of variable changes within the group was calculated based on the Friedman test; ^*∗∗*^*P* value of frequency, duration, and severity of differences in week 0 and week 12 between groups was calculated based on the Kruskal-Wallis test. **Group 1:** the poly-herbal formulation capsule 1500 mg (two capsules of 750 mg) three times a day,  + Depakine® tablet 200 mg twice a day + Amitriptyline tablet 10 mg once a day; **Group 2:** Depakine® tablet 200 Mg twice a day + Amitriptyline 10 mg once a day + the poly-herbal formulation placebo capsule three times a day; **Group 3:** the poly-herbal formulation capsule 1500 mg (two capsules of 750 mg) three times a day + Depakine® placebo tablet twice a day.

**Table 3:** The effects of different interventions on HIT-6 and FD scores at week 0 and week 12.

Group no.	Score		*P* value^*∗*^	∆(W12–W0) score
	*HIT- 6*			
	Week 0	Week 12		
1	65.86 ± 2.43	56.17 ± 4.59	*P* < 0.001	9.69 ± 4.62
2	65.62 ± 2.02	55.59 ± 4.39	*P* < 0.001	9.69 ± 4.39
3	66.45 ± 2.75	56.27 ± 4.96	*P* < 0.001	10.18 ± 4.46
*P* value^*∗∗*^	0.88	0.87		*P*=0.40

	*FD score*			
	Week 0	Week 12		
1	8.86 ± 2.66	4.69 ± 1.98	*P* < 0.001	4.17 ± 2.01
2	8.43 ± 2.48	5.95 ± 1.79	*P* < 0.001	2.47 ± 1.56
3	8.90 ± 2.02	5.13 ± 1.85	*P* < 0.001	3.77 ± 1.77
*P* value^*∗∗*^	0.88	0.11		*P*=0.06

Data were presented as mean and standard deviation (SD). ^*∗*^*P* value was calculated by the Wilcoxon test; ^*∗∗*^*P* value was calculated by Kruskal-Wallis. **Group1:** the polyherbal formulation capsule^*∗*^ 1500 mg (two capsules of 750 mg) three times a day, + Depakine® tablet 200 mg twice a day + Amitriptyline tablet 10 Mg once a day; **Group2:** Depakine® tablet 200 Mg twice a day + Amitriptyline 10 mg once a day + the polyherbal formulation placebo capsule^*∗*^ three times a day; **Group3:** the polyherbal formulation capsule 1500 mg (two capsules of 750 mg) three times a day + Depakine® placebo tablet twice a day.

**Table 4:** Correlation of duration, severity, frequency of MH attacks and HIT-6, at the end of intervention (week 12) with a score of FD at the end of intervention according to Spearman's Rho test.

Group NO	Duration	Severity	Frequency	HIT-6
Group 1	*ρ* = 0.28 *P*=0.19	*ρ* = 0.02 *P*=0.91	*ρ* = −0.4 *P*=0.83	*ρ* = 0.29 *P*=0.16

Group 2	*ρ* = 0.14 *P*=0.51	*ρ* = 0.45 *P*=0.03	*ρ* = 0.31 *P*=0.14	*ρ* = 0.47 *P*=0.02

Group 3	*ρ* = 0.55 *P*=0.007	*ρ* = 0.57 *P*=0.005	*ρ* = 0.41 *P*=0.05	*ρ* = 0.66 *P*=0.001

**Group 1:** the polyherbal formulation capsule*∗* 1500 mg (two capsules of 750 mg) three times a day, + Depakine® tablet 200 mg twice a day + Amitriptyline tablet 10 mg once a day; **Group 2:** Depakine® tablet 200 Mg twice a day + Amitriptyline 10 mg once a day + the polyherbal formulation placebo capsule*∗* three times a day; **Group 3:** the polyherbal formulation capsule 1500 mg (two capsules of 750 mg) three times a day + Depakine® placebo tablet twice a day.

**Table 5:** Some reported gastrointestinal actions of the polyherbal formulation ingredients.

Ingredients	Scientific name	Important phytochemical	Actions on GI
Damask	*Rosa damascena*	Geraniol, citronellol, nerol, phenylethyl alcohol [76].	Laxative, astringent [77] increases the lower oesophageal sphincter tonicity and anti-reflux [64,78].
Coriander	*Coriandrum sativum*	Linalool, *α*-pinene, limonene, gamma-terpinene, camphor, geraniol, geranylacetate [79].	Carminative, improving gastric juices secretion, spasmolytic. Approved for dyspeptic complaints, Loss of appetite and complaints of the upper abdomen [65,80,81].
Ginger	*Zingiber officinalis*	Zingiberone, curcumene, bisrabolone, *α*-pinene, *β*-pinene, limonene, p-cymene, *α*-terpineol, and verticiole [82,83].	Prokinetic, antiulcer, carminative, anti-dyspeptic, Useful for nausea relief, decrease pressure on the lower oesophagal sphincter, reduce intestinal cramping, and stimulate antral contractions in healthy people individuals and gastrotonic [63,67,83].
Indian gooseberry	*Phyllantus embelica*	Phyllantadine, ascorbic acid, seasamine, beta carotene, estradiol, astagallin, lupenol, kaemferole, phyllanthine, chebulanin [84–87].	It increases the lower esophagal sphincter tonicity, anti-reflux, prokinetic and laxative activities, gastrotonic [41,88–90].

## Data Availability

The datasets generated during and/or analyzed during the current study are available from the corresponding author on reasonable request.

## Additional Points

In accordance with the existing laws in the research department of the University, human rights have been observed.
